# Flame Inhibition and Charring Effect of Aromatic Polyimide and Aluminum Diethylphosphinate in Polyamide 6

**DOI:** 10.3390/polym11010074

**Published:** 2019-01-05

**Authors:** Haisheng Feng, Yong Qiu, Lijun Qian, Yajun Chen, Bo Xu, Fei Xin

**Affiliations:** 1 School of Materials Science and Mechanical Engineering, Beijing Technology and Business University, Beijing 100048, China; fhsbtbu@163.com (H.F.); btbuqy@126.com (Y.Q.); chenyajun@th.btbu.edu.cn (Y.C.); xubo@btbu.edu.cn (B.X.); xinfei@th.btbu.edu.cn (F.X.); 2 Engineering Laboratory of non-Halogen Flame Retardants for Polymers, Beijing 100048, China; 3 Beijing Key Laboratory of Quality Evaluation Technology for Hygiene and Safety of Plastics, Beijing 100048, China; 4 Fire Protection Engineering Department, China People’s Police University, Hebei 065000, China; 5 School of Materials Science and Engineering, Beijing Institute of Technology, Beijing 100081, China

**Keywords:** flame retardant, charring, mechanical properties, polyamide 6, polyimide, synergistic effect

## Abstract

An aromatic macromolecular polyimide (API) was synthesized and characterized, and used as a synergistic charring flame retardant in glass fiber reinforced polyamide 6 (GF/PA6). API and aluminum diethylphosphinate (ADP) exhibited better flame inhibition behavior and synergistic charring flame retardant behavior compared with ADP alone. The 5%API/7%ADP/GF/PA6 sample achieved the lower peak value of the heat release rate (pk-HRR) at 497 kW/m^2^ and produced higher residue yields of 36.1 wt.%, verifying that API and ADP have an outstanding synergistic effect on the barrier effect. The API/ADP system facilitated the formation of a carbonaceous, phosphorus and aluminum-containing compact char layer with increased barrier effect. FTIR spectra of the residue and real-time TGA-FTIR analysis on the evolved gases from PA6 composites revealed that API interacted with ADP/PA6 and locked in more P–O–C and P–O–Ar content, which is the main mechanism for improving flame inhibition and charring ability. In addition, the API/ADP system improved the mechanical properties and corrosion resistance of GF/PA6 composites compared to ADP alone.

## 1. Introduction

Polyamide 6 (PA6) is a significant engineering resin that is widely used in electronics, automotive, high-speed railway, aircraft, household products and so on, because of its excellent toughness, good self-lubrication and thermal resistance [1,2]. However, PA6 is a flammable material with dripping during combustion, which poses potential fire risks. Thus, PA6 requires flame retardant properties for its commercial application.

In the past decades, brominated flame retardants (BFRs), which are a broad group of chemicals have been added to polyamide due to their cost-effectiveness, generality, minimal effect on mechanical properties and their significant potential for delaying ignition. However, studies on the toxicity of BFRs have led to serious health and environmental concerns and the usage of BFRs has been limited worldwide [3,4,5]. Thus, as an alternative to BFRs, the halogen-free flame retardant system has become the focus of interest. In current studies on PA6 with halogen-free flame retardant systems, research teams have mainly developed polyamide materials with high flame retardant performance by using the following four methods: (1) constructing an efficient flame retardant system by blending different components and utilizing the component synergistic effect [6,7,8,9,10,11,12,13,14]; (2) obtaining a new flame retardant system by bonding different flame retardant groups into one molecule [15,16,17,18,19,20,21]; (3) designing novel flame retardant chemical structures [22,23,24]; and (4) preparing intrinsically flame retardant polyamide [25,26]. However, traditional halogen-free flame retardants for polyamide, such as melamine polyphosphate (MPP) [27] and melamine cyanurate (MCA) [28], have been unable to meet commercial demands for improved physical-mechanical properties because of their low flame retardant efficiency. In recent years, aluminum diethylphosphinate (ADP) as a highly efficient phosphorus-based flame retardant has provided a new industrial solution for flame-retarded PA6 because of its eco-friendliness, excellent electrical properties and good water resistance [29]. Generally, PA6 composites with 13–15% ADP can achieve better flame retardant effects (UL94 V-0) [30]. However, due to its acidity and compatibility with the matrix, these amounts of ADP in composites lead to corrosion of the processing machines in long-term applications, and deterioration of the physical-mechanical properties, which calls for further improvements in ADP application in PA materials.

**Scheme 1:**
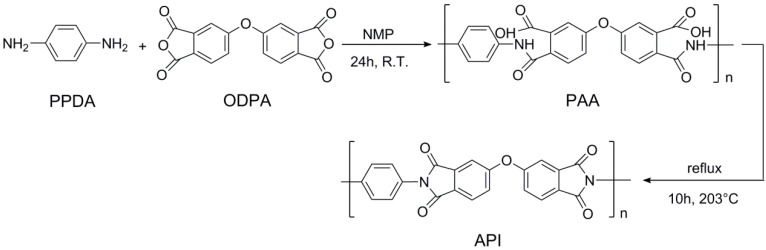
Synthesis of API compound.

The charring behavior in flame retardant system is of great importance because it not only locks in the combustible components and reduces flammable gas release but also generates barrier protection effects on substrate [31,32,33,34,35]. In this thesis, a linear polyether aromatic polyimide (API, shown in Scheme 1), was designed and synthesized to facilitate the charring effect of PA6 composites and construct a better flame retardant system using ADP/PA6. The charring effect, flame inhibition, mechanism of pyrolysis and the physical-mechanical properties of API/ADP in glass fiber reinforced PA6 were investigated and discussed.

## 2. Experimental Methods

### 2.1. Materials

5,5′-Oxybis (isobenzofuran-1,3-dione) (ODPA) was supplied by Illinois Ark Pharm Co., Ltd. (Libertyville, IL, USA). USA. p-Phenylenediamine (PPDA) and methanol were acquired from Shanghai Macklin Biochemical Co., Ltd. (Shanghai, China). N-methylpyrrolidinone (NMP) was purchased from Shanghai Aladdin industrial Co., Ltd. (Shanghai, China). NMP was distilled and then stored with 4Å molecular sieves. Before use, dianhydride and diamine were dried under primary vacuum at 100 °C for 12 h. PPDA was recrystallized twice. All solvents and reagents were analytical class.

The granuliform polyamide 6 (1013B) was supplied by Sinopec Shijiazhuang Refining & Chemical Company (Shijiazhang, Hebei, China). Aluminum diethylphosphinate (ADP, OP935) was purchased from Clariant Chemical Co., Ltd. (Shanghai, China). Antioxidant 1010 and antioxidant 168 were purchased from Sinopharm Chemical Reagent Co., Ltd. (Shanghai, China). Short glass fiber (GF, 568H) was produced by Jushi Group Co., Ltd. (Jiaxing, Zhejiang, China).

### 2.2. Synthesis of API Compound

In an ice bath, PPDA (21.63 g 0.2 mol) was completely dissolved in 756 g NMP at nitrogen atmosphere. Then, ODPA (62.04 g, 0.2 mol) was added evenly into the solution in 1 h. The reaction mixture was stirred for 24 h at room temperature to prepare polyamic acid. Then, at the boiling point of NMP (b.p. 203 °C), the polyamic acid was dehydrated for 10 h to obtain API powder. After being cooled to room temperature, the final yellow powder was filtered and washed 3 times in methanol, and then dried for 4 h under vacuum at 180 °C. The reaction formula for preparing API is shown in Scheme 1. Fourier Transform Infrared (FTIR, Nicolet iZ10, Thermo Fisher Scientific, Inc., Madison, WI, USA) (KBr, cm^−1^): 1778 (asymmetrical C=O stretch vibration) and 1720 (symmetrical C=O stretch vibration), 1357 (C–N stretch vibration), 695 (C=O bend vibration). Solid-state ^13^C CP/MAS-TOSS NMR (Bruker Daltonics Inc. Karlsruhe, Germany) (δ, ppm): 164.96 (C=O), 159.62 (C–O–), 138.66 (–O–CH–C–C=O), 131.24 (C–N), 129.77 (CH–C–N), 126.35 (CH–CH–C–C=O), 123.04 (CH–CH–C–C=O), 120.21 (CH–CH–C–O–), 113.88 (C–CH–C–O–). Elemental analysis (C_22_H_12_N_2_O_5_)*_n_*:C: 68.56 ± 0.057% (calculated value: 68.74%); H: 3.06 ± 0.028% (calculated value: 3.15%); N: 7.38 ± 0.071% (calculated value: 7.29%). The data from the FTIR and ^13^C NMR are illustrated in Figures S1 and S2, respectively.

### 2.3. Preparation of PA6 Composites

Firstly, the materials, PA6, GF, API, ADP, antioxidant 1010 and antioxidant 168 were dried in a vacuum oven at 120 °C for 3.5 h. Secondly, according to their different ratios, as described in Table 1, all the above materials were added into a torque rheometer. The mixture was heated to 240 °C and blended for 10 min. Finally, the blends were pressed to 3 mm thick standard specimens at 240 °C under 50 MPa. Then, they were cut into test specimens with a standard size.

### 2.4. Characterization

#### 2.4.1. Structural Characterization

FTIR spectra were recorded with an iZ10 spectrophotometer (Nicolet) with a resolution of 6 cm^−1^ (KBr pellets).

Solid-state ^13^C CP/MAS-TOSS NMR spectrum was obtained using an AVANCE III HD 700MHz NMR spectrometer (Bruker, Karlsruhe, Germany) running at a spinning speed of 12 kHz and using a Bruker probe head (PA BBO 700S3 BB-H-D-05 Z) equipped with a 3.2 mm MAS assembly.

Element contents were investigated via a Vario EL elemental analyzer (Elementar Analysensysteme GmbH, Inc, Langenselbold, Germany) with a reduction temperature of 700 °C and a combustion temperature of 950 °C. The results were the average of the 2 times repeated tests.

#### 2.4.2. Limited Oxygen Index (LOI) Measurement

LOI value measurements were conducted using a FTT (Fire Testing Technology) Dynisco LOI instrument (Fire Testing Technology, London, UK) according to ASTM D2863 (sample dimension: 100.0 mm × 6.5 mm × 3.2 mm).

#### 2.4.3. Vertical Burning Test

The UL94 flammability classification was measured via a FTT 0082 instrument (Fire Testing Technology, London, UK) according to ASTM D3801 with sample dimensions of 125.0 mm × 12.7 mm × 3.2 mm.

#### 2.4.4. Cone Calorimeter Test

Combustion behavior was studied using a FTT cone calorimeter according to ISO 5660 at an external heat flux of 50 kW/m^2^ (sample dimension: 100.0 mm × 100.0 mm × 3.0 mm).

#### 2.4.5. Scanning Electron Microscope (SEM) and Energy Dispersive X-Ray Spectroscopy (EDX)

The micro morphology after the cone calorimeter test was observed with a Phenom^TM^ Pro SEM (Phenom World, Eindhoven, Netherlands) equipped with EDX with 10 kV for SEM and 15 kV for EDX.

#### 2.4.6. Thermogravimetry—Fourier Transform Infrared Spectroscopy

The thermogravimetric analysis (TGA) was carried out via a STA 8000 simultaneous thermal analyzer (Perkin Elmer, Waltham, MA. USA). All the samples were heated from 50 °C to 800 °C at the rate of 20 °C/min in N_2_ atmosphere. The real-time FTIR spectra of pyrolysis gas from the TGA were collected via Frontier FTIR spectrometer (Perkin Elmer, Waltham, MA. USA). The temperature of the sample cell in the spectrometer was kept at 280 °C constantly. The pyrolysis processes of the samples were detected via TGA-FTIR in real-time.

#### 2.4.7. Tensile and Unnotched Charpy Impact Test

Tensile strength was tested using a CMT6004 electromechanical universal testing machine (MTS systems Co., Ltd. Shanghai, China) according to ISO 527-1 at the speed of 5 mm/min with a gauge length of 80 mm, a width of 10 mm and a thickness of 4 mm. The results were the average of 5 repeated tests. Unnotched impact strength was tested by a XJZ-50 digital impact test machine (Cheng De testing machine Co., Ltd. Chengde, China) according to ISO 179-1 with a 5 J pendulum. The results were the average of 5 repeated tests (sample dimension: 80 mm × 10 mm × 4 mm).

## 3. Results and Discussion

### 3.1. Synthesis of API

As shown in Figure S1, the FTIR spectrum exhibited four characteristic imide group absorptions, which are known as imide group I, II, III and IV bands [36,37]: imide I band at 1778 cm^−1^ was asymmetrical C=O stretch vibration; imide II band at 1720 cm^−1^ was symmetrical C=O stretch vibration; imide III band at 1357 cm^−1^ was C-N stretch vibration; and imide IV band at 695 cm^−1^ was C=O bend vibration. All the four imide bands corresponded with the typical absorptions of the imide group. The typical chemical shifts at 164.96 ppm and 159.62 ppm in solid-state ^13^C NMR data and spectrum (Figure S2) represented C=O and C–O– structures [38]. The other typical chemical shifts corresponded to the structures of C–C or C–N bonds including 138.66 (–O–CH–C–C=O), 131.24 (C–N), 129.77 (CH–C–N), 126.35 (CH–CH–C–C=O), 123.04 (CH–CH–C–C=O), 120.21 (CH–CH–C–O–) and 113.88 (C–CH–C–O–). All chemical shifts in the NMR spectrum coincided well with the API chemical structure. API elemental analysis results were nearly equal to the calculated value (C_22_H_12_N_2_O_5_)*_n_*, further proving the successful preparation of API.

### 3.2. Thermal Degradation Behaviors of Flame Retardant PA6 Composites

**Figure 1:**
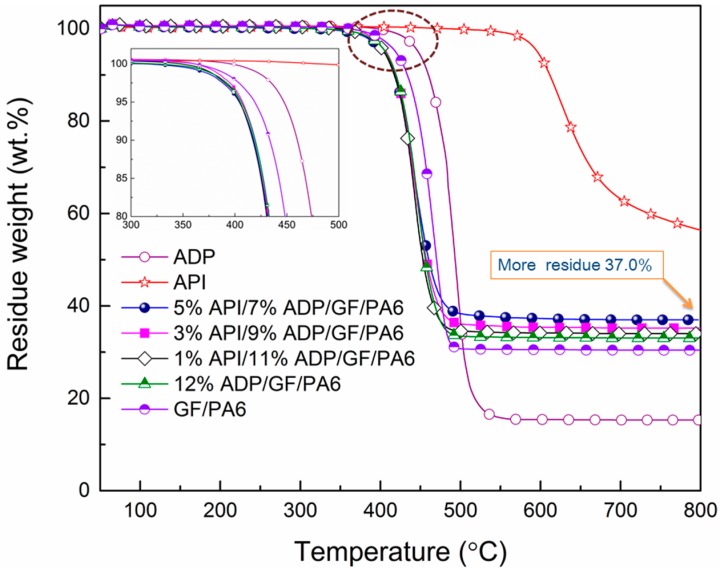
TGA curves for API, ADP and PA6 composites.

The thermal degradation curves of API, ADP, GF/PA6, ADP/GF/PA6 and API/ADP/GF/PA6 tested by TGA in N_2_ are presented in Figure 1 and the typical TGA data in curves are listed in Table 2. API and ADP showed a higher onset decomposition temperature (*T*_d,2%_) and maximum decomposition temperature (*T*_d,max_) than all the flame retardant PA6 composites. Although API and ADP possess higher thermal stability than GF/PA6, API/ADP still endowed API/ADP/GF/PA6 composites with a lower onset decomposition temperature in contrast to that of GF/PA6. The flame retardant API/ADP induced earlier decomposition of the PA6 matrix. Another effect of API/ADP was the increasing residue yields of API/ADP/GF/PA6 composites in the TGA test, as shown in Table 2. With the increasing ratio of API in API/ADP/GF/PA6 composites with constant total amounts of flame retardants, the residual yields of API/ADP/GF/PA6 composites at 800 °C also gradually increased, which indicated that API enabled a better charring effect from API/ADP/GF/PA6 during thermal degradation. API/ADP/GF/PA6 demonstrated higher condensed-phase flame retardant efficiency compared to ADP/GF/PA6 because of the added API. Further evidence will be provided in the subsequent discussion.

### 3.3. Flame Retardancy

In order to investigate the working mode of API with ADP, the flame retardant properties of the prepared samples were detected by the LOI value, UL94 testing and cone calorimeter instruments.

According to the LOI and UL94 rating results shown in Table 3, in comparison to the other prepared samples, 5 wt % API sustained a V-0 rating from the UL94 when the total addition of the API/ADP flame retardant system in PA6 composites was 12 wt %, indicating that 5%API/7%ADP contributed to a high flame retardant performance and minimized the corrosion of ADP.

The cone calorimeter test, which is relatively close to the condition of real fire discloses more details of the burning behaviors and effectively analyzes the flame retardancy of polymer composites during combustion [39]. The typical parameters of the prepared samples obtained from the cone calorimeter are listed in Table 4, include time to ignition (TTI), heat release rate (HRR), total heat rate (THR), residue, total smoke release (TSR), average CO yields (av-COY), average CO_2_ yields (av-CO_2_Y), and average effective heat of combustion (av-EHC).

TTI is the time for the specimen to be ignited by the electric spark of the cone calorimeter. GF/PA6 took just 33 s to be ignited, whereas the TTI values for specimens with API/ADP were more than 42 s, implying that the addition of API reduced the flammability of PA6 composites.

**Figure 2:**
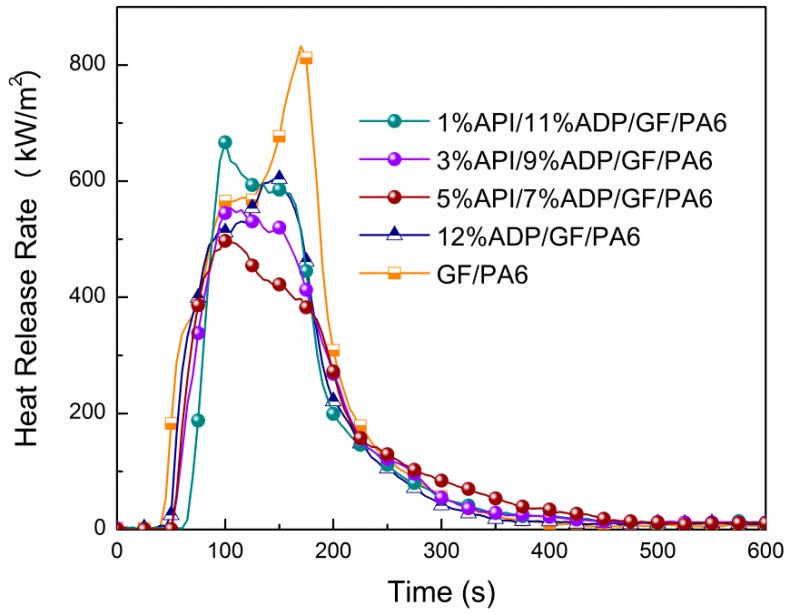
HRR curves of PA6 composites in the cone calorimeter test.

The HRR curves, shown in Figure 2, demonstrated that 12% ADP led to a lower peak value of HRR compared to GF/PA6. Otherwise, as shown in Table 4, the peak HRR value(pk-HRR) for 5%API/7%ADP/GF/PA6 was just 497 kW/m^2^, which is obviously lower than the value of 604 kW/m^2^ for 12%ADP/GF/PA6 and 817 kW/m^2^ for GF/PA6. Meanwhile, all flame retardant PA6 composites with incorporated API showed a later heat release, which implied that API generated matrix decomposed at a later time. At the early stage of combustion, 5%API in 5%API/7%ADP/GF/PA6 interrupted the increased combustion intensity and formed the first relatively low HRR. With the development of combustion, API also obviously decreased the pk-HRR of 5%API/7%ADP/GF/PA6. Additionally, all the specimens burnt out almost simultaneously. The results indicated that API incorporated into ADP/GF/PA6 shortened the combustion time and the API/ADP system exerted a better flame inhibition effect during combustion than ADP did alone in GF/PA6.

With the increasing ratio of API in API/ADP system, THR values gradually decreased when the total amount of flame retardant in GF/PA6 was constant, which verified that API worked together with ADP to weaken the burning intensity of PA6 composites. The API/ADP system generated a synergistic flame retardant effect in contrast to ADP alone in GF/PA6.

The residue yields of API-containing PA6 composites appeared to increase with an increase in the added amounts of API, as shown in Table 4, which implies that API enabled a better charring effect from API/ADP/GF/PA6 during combustion. The increase in residue yields led to the reduced release of fuels, which indicates that API/ADP/GF/PA6 facilitated the flame inhibition efficiency in the condensed-phase compared to ADP/GF/PA6 because of the incorporation of API.

In addition, TSR, av-COY and av-CO_2_Y reveal more detailed information about the combustion of the materials. In comparison with ADP/GF/PA6, there was a slight decrease in av-CO_2_Y values and TSR values for samples with API/ADP. Moreover, the av-EHC values of samples with the API/ADP system decreased compared to those of ADP/GF/PA6 and GF/PA6, signifying that incomplete burning was enhanced, the flammable gas release was reduced and the gas-phase burning reaction was more strongly inhibited. Thereby, it was inferred that the API/ADP system facilitated the flame retardant efficiency in the gas phase, although slight variations in TSR, av-COY and av-CO_2_Y were detected.

### 3.4. Macroscopic and Microscopic Morphologies of Residues

**Figure 3:**
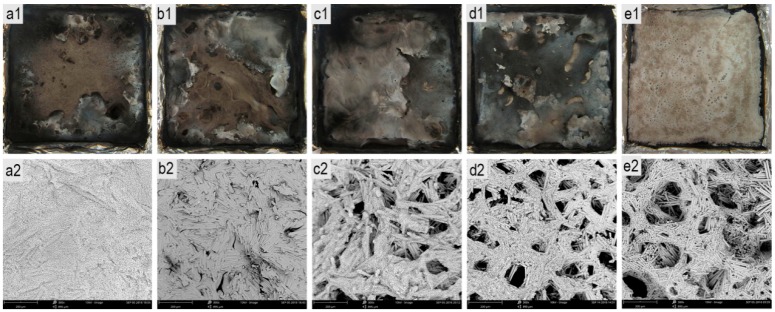
Digital photos (**a1**–**e1**) and SEM photos (**a2**–**e2**) of residue from cone calorimeter tests (**a1**,**a2**) 5%API/7%ADP/GF/PA6; (**b1**,**b2**) 3%API/9%ADP/GF/PA6; (**c1**,**c2**) 1%API/11%ADP/GF/PA6; (**d1**,**d2**) 12%ADP/GF/PA6; (**e1**,**e2**) GF/PA6.

The macroscopic digital photos and microscopic SEM images of the residues of PA6 composites from the cone calorimeter test shown in Figure 3, present further details about the combustion behavior of the prepared samples. As shown in Figure 3(e1), the residue from GF/PA6, appears white and exhibited a porous, loose and broken state, implying that the burnt GF/PA6 residue only retained glass fiber and the untreated GF/PA6 exhibited the lowest charring ability. In Figure 3(a1), more residues, possessing greater rigidity, remained because of the API/ADP added into GF/PA6, signifying that the interaction between API/ADP and the matrix facilitated better charring effects.

The morphological variation in the residues of the prepared samples was more clearly observed in the SEM photos. In Figure 3(e2) the residue of GF/PA6 displayed the accumulation morphology of glass fiber without the existence of carbonaceous content, thereby proving that GF/PA6 had the worst charring ability. In Figure 3(d2), the burnt 12%ADP/GF/PA6 had residues with carbonaceous contents combined with glass fiber, although some holes and cracks were also observed, implying that ADP was responsible for the charring ability to some extent. In Figure 3(a2,b2,c2), with the increasing API fractions, a compact residue gradually formed and the holes within the residue gradually disappeared after the combustion of API/ADP/GF/PA6. In the case of 5%API/7%ADP/GF/PA6, the residue surface was completely covered by dense carbonaceous content with little glass fiber and few holes and cracks observed, signifying the barrier effect on the exchange of oxygen and flammable gas during combustion. From the combined cone calorimeter data and microscopic SEM image of the residue from 5%API/7%ADP/GF/PA6, as shown in Figure 3(a2), it was inferred that 5%API/7%ADP facilitated the formation of compact, strong and thick residue with few holes and cracks which sealed the fuel inside and hindered the heat and oxygen supply, thus weakening the combustion process, which also signified the improvement in the charring ability and the condensed-phase flame retardant efficiency.

**Figure 4:**
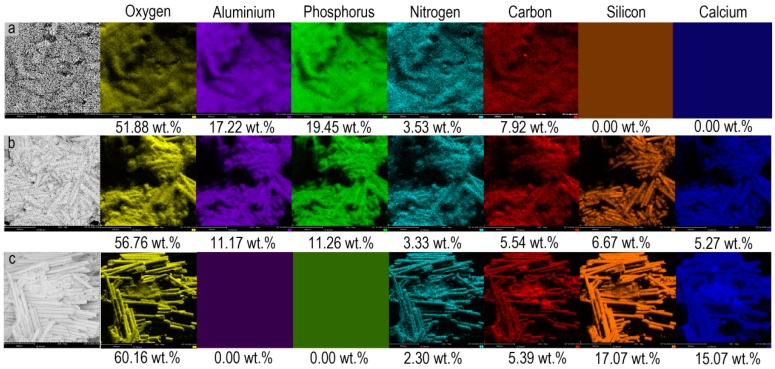
EDX scanned photos of surface residue from cone calorimeter tests (**a**) 5%API/7%ADP/GF/PA6; (**b**) 12%ADP/GF/PA6; (**c**) GF/PA6.

The composition of the elements in the residue was observed with EDX and provided additional persuasive evidence that 5%API/7%ADP/GF/PA6 exerted a high flame retardant effect. Figure 4 demonstrates the element contents of the residues of GF/PA6, 12%ADP/GF/PA6 and 5%API/7%ADP/GF/PA6. Calculation of the element compositions showed that 5%API/7%ADP/GF/PA6 had the highest carbon (C), nitrogen (N), aluminum (Al) and phosphorus (P) mass ratios of 7.92 wt %, 3.53 wt %, 17.22 wt % and 19.45 wt %, respectively, in comparison with GF/PA6, and 12%ADP/GF/PA6. Thereby, it was implied that ADP interacted with API to lock more carbonaceous and phosphorous fragments in the residue and enrich Al elements on the residue surface, resulting in less flammable gas, and stronger and denser residue for isolating oxygen and heat. Therefore, during combustion, the appropriate proportion of API and ADP effectively inhibits the burning intensity and facilitates the charring process.

### 3.5. FTIR Analysis of Residues

**Figure 5:**
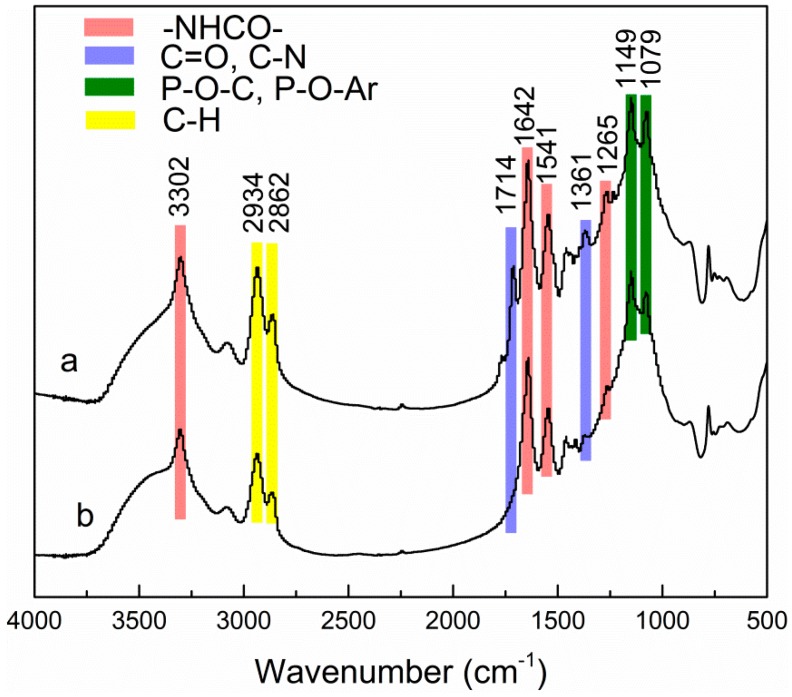
FTIR spectra of PA6 composites from cone tests (**a**) 5%API/7%ADP/GF/PA6; (**b**) 12%ADP/GF/PA6.

In order to further clarify the joint working mechanism of API and ADP, the FTIR spectra of 5%API/7%ADP/GF/PA6 and 12%ADP/GF/PA6 were detected and used to analyze the differences in their chemical structures, see Figure 5. The spectra of residues from 5%API/7%ADP/GF/PA6 and 12%ADP/GF/PA6 were quite similar, but some subtle differences were still perceived. The peaks at 1714 cm^−1^ and 1361 cm^−1^, representing C-O and C-N structures, were found only on the spectrum of the residue from 5%API/7%ADP/GF/PA6. In addition, the spectra of residues from 5%API/7%ADP/GF/PA6 and 12%ADP/GF/PA6 preserved several absorption peaks at 3302 cm^−1^, 1642 cm^−1^ and 1541 cm^−1^ (–NHCO–), 2934 cm^−1^ and 2862 cm^−1^ (C–H), 1149cm^−1^ and 1079cm^−1^ (P–O–Ar and P–O–C). Though observed in both the residue from 5%API/7%ADP/GF/PA6 and that from 12%ADP/GF/PA6, these peaks showed slightly stronger intensity on the spectrum of the residue from 5%API/7%ADP/GF/PA6 in comparison with that from 12%ADP/GF/PA6. More chemical groups containing N, P and C were found in the residue from 5%API/7%ADP/GF/PA6, which proved that the joint work of API and ADP resulted in more carbonaceous, phosphorus and nitrogenous content in the residue, which further testified that API/ADP played a crucial role in facilitating the charring ability in PA6 composite.

### 3.6. Real-Time TGA-FTIR Analysis on the Evolved Gases from PA6 Composites

**Figure 6:**
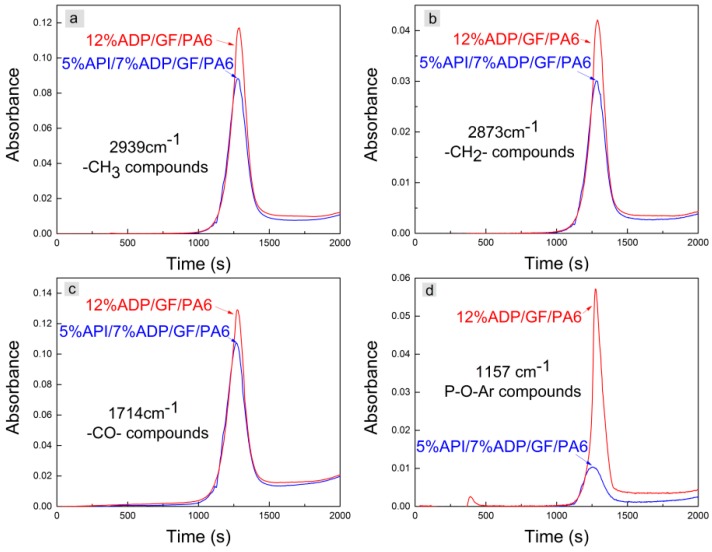
Intensity-time curves of typical absorption peak in real-time FTIR spectra of TGA pyrolysis gases of 5%API/7%ADP/GF/PA6 and 12%ADP/GF/PA6. (**a**) –CH_3_ compounds; (**b**) –CH_2_– compounds; (**c**) –CO– compounds; (**d**) P–O–Ar compounds.

Real-time TGA-FTIR was carried out to study the volatiles of the samples. Before the test, the comparability of the data was ensured by testing the samples using the same mass. Some typical FTIR absorption peaks were selected to track the change of their intensity with time online. As shown in Figure 6, the same characteristic peaks were found on both the spectrum of the volatiles of 5%API/7%ADP/GF/PA6 and that of 12%ADP/GF/PA6: 2939 cm^−1^ (–CH_3_), 2873 cm^−1^ (–CH_2_–), 1714 cm^−1^ (–CO–) and 1157 cm^−1^ (P–O–Ar), but these absorption peaks manifested visibly lower intensity on the spectrum of the volatiles of 5%API/7%ADP/GF/PA6 in contrast to that of 12%ADP/GF/PA6. Moreover, the absorption peak at 1157 cm^−1^ on the two spectra showed the greatest difference in intensity. The results confirmed that the interaction between API and ADP inhibited the release of the main gas fragments, thus reducing the generation of fuels. Meanwhile, when there were more contents containing P and C, which were preserved in the condensed phase to form compact residue, heat and oxygen were prevented from feeding the flame, and the release of fuels was also hindered. Therefore, a reliable conclusion was reached that API and ADP jointly worked to weaken the combustion process and facilitate the charring process.

### 3.7. Mechanical Properties of PA6 Composites

Like most advanced functional materials, advanced PA6 materials also seek balance in their application performance, especially for the most mechanical properties and flame retardancy [40]. The tensile strength and impact strength of PA6 composites are shown in Table 5. Compared with GF/PA6, the tensile strength and impact strength of 12%ADP/GF/PA6 decreased by 17.1% and 24.5%, respectively. However, the tensile strength and impact strength of the 5%API/7%ADP/GF/PA6 system were stronger than those of 12%ADP/GF/PA6 and close to those of GF/PA6. Moreover, compared with GF/PA6, the tensile strain at break of 5%API/7%ADP/GF/PA6 increased by 53.6%. The results implied that the incorporation of API in flame retardant system and replacing some of the ADP, reduced the deterioration in the mechanical properties of ADP/GF/PA6. The API/ADP flame retardant system resulted in enhancement of tensile strength and impact strength.

## 4. Conclusions

A macromolecular compound API was synthetized and characterized. API and ADP were applied in glass fiber reinforced polyamide 6 to explore more effective flame retardant systems. GF/PA6 with 5%API7%ADP decreased the peak value of the heat release rate (pk-HRR) to 497 kW/m^2^ and boosted the residue yields to 36.1 wt %. The interactions between API and ADP/GF/PA6 locked more content containing C and P and enriched Al elements on the residue surface. The locking of most of the P–O–C and P–O–Ar groups and abundance of Al elements created a strong barrier effect, resulting in higher flame retardant efficiency and charring ability with the addition of the amounts of 5% API into 7%ADP/GF/PA6. The compact and strong char layer, hinders oxygen and heat from penetrating inside and inhibits flammable gas from being emitted, this enables better flame retardancy in GF/PA6. In addition, tensile strength and impact strength revealed that the incorporation of API to replace some of the ADP improved the mechanical properties of ADP/GF/PA6. As a kind of aromatic polyimide, API is expected to become an effective charring agent in engineering plastics.

## Figures and Tables

**Table 1:** Formulae of flame retardant PA6 composites.

Samples	PA6 (wt %)	GF (wt %)	ADP (wt %)	API (wt %)	Antioxidant1010 (wt %)	Antioxidant168 (wt %)
1%API/11%ADP/GF/PA6	57.4	30	11.0	1.0	0.4	0.2
3%API/9%ADP/GF/PA6	57.4	30	9.0	3.0	0.4	0.2
5%API/7%ADP/GF/PA6	57.4	30	7.0	5.0	0.4	0.2
12%ADP/GF/PA6	57.4	30	12.0	0.0	0.4	0.2
GF/PA6	69.4	30	0.0	0.0	0.4	0.2

**Table 2:** Typical TGA data for API, ADP and PA6 composites.

Samples	*T*_d,2%_ (°C)	*T*_d,max_ (°C)	Residue at 800 °C (%)
API	577	620	56.2
ADP	431	493	15.3
1%API/11%ADP/GF/PA6	386	441	34.0
3%API/9%ADP/GF/PA6	391	441	35.2
5%API/7%ADP/GF/PA6	383	444	37.0
12%ADP/GF/PA6	387	447	33.0
GF/PA6	399	468	30.4

**Table 3:** LOI value and UL94 rating of PA6 composites.

Samples	LOI (%)	UL94			
		av-t_1_ (s)	av-t_2_ (s)	Dripping	Rating
1%API/11%ADP/GF/PA6	35.4	1.4	1.7	NO	V-0
3%API/9%ADP/GF/PA6	34.7	1.2	4.1	NO	V-0
5%API/7%ADP/GF/PA6	34.2	2.2	4.8	NO	V-0
12%ADP/GF/PA6	34.0	1.2	2.1	NO	V-0
GF/PA6	23.1	34.2	--	YES	No rating

**Table 4:** Typical parameters of PA6 composites in the cone calorimetry test.

Samples	TTI (s)	THR (MJ/m^2^)	pk-HRR (kW/m^2^)	Residue (wt %)	av-EHC (MJ/kg)	av-COY (kg/kg)	av-CO_2_Y (kg/kg)	TSR (m^2^/m^2^)
1%API/11%ADP/GF/PA6	57 ± 1	81.9 ± 1.3	667 ± 18	31.5 ± 0.3	28.8 ± 0.4	0.15 ± 0.01	2.12 ± 0.18	1858 ± 49
3%API/9%ADP/GF/PA6	46 ± 1	81.8 ± 0.5	554 ± 22	32.5 ± 0.3	28.4 ± 0.5	0.15 ± 0.02	2.13 ± 0.22	1872 ± 70
5%API/7%ADP/GF/PA6	44 ± 1	80.6 ± 2.1	497 ± 15	36.1 ± 0.4	28.2 ± 0.4	0.14 ± 0.01	2.17 ± 0.13	1821 ± 54
12%ADP/GF/PA6	42 ± 1	84.3 ± 0.8	604 ± 21	31.0 ± 0.5	29.6 ± 0.6	0.15 ± 0.02	2.19 ± 0.20	1889 ± 40
GF/PA6	33 ± 2	116.5 ± 1.7	817 ± 36	30.1 ± 0.4	36.4 ± 0.9	0.03 ± 0.01	2.84 ± 0.28	739 ± 68

**Table 5:** Mechanical properties of PA6 composites.

Samples	Tensile Strength (MPa)	Impact Strength (kJ/m^2^)	Tensile Strain at Break (%)
1%API/11%ADP/GF/PA6	71.5 ± 1.9	27.0 ± 1.1	3.7 ± 0.3
3%API/9%ADP/GF/PA6	78.8 ± 0.6	27.2 ± 1.5	3.8 ± 0.4
5%API/7%ADP/GF/PA6	83.5 ± 0.6	28.5 ± 1.5	4.3 ± 0.4
12%ADP/GF/PA6	70.0 ± 3.4	20.6 ± 1.2	2.5 ± 0.4
GF/PA6	84.4 ± 1.4	27.3 ±2.2	2.8 ± 0.4

## Supplementary Materials

The following are available online at http://www.mdpi.com/2073-4360/11/1/74/s1, Figure S1. Figure S1. FTIR spectrum of API, Figure S2. Figure S2. Solid-state 13C NMR spectrum of API.

## Fundings

This work was funded by the Beijing Municipal Training Program for Great Wall Scholars (No. CIT&TCD20180312) and Young Top-notch Talents Support Project of High-level Teachers in Beijing Municipal Universities in the Period of the 13th Five-year Plan (No. CIT&TCD201704040).
